# Navigating challenges in Vietnamese enterprises: An examination of the interplay between environmental regulations, organizational innovation, resilience, learning support, and performance

**DOI:** 10.1371/journal.pone.0313075

**Published:** 2024-12-17

**Authors:** Thang Nam Huynh, Phuong Van Nguyen, Ngan Phi Doan, Khoa Tien Tran, Tien Canh Nguyen

**Affiliations:** 1 Center for Public Administration, International University- Vietnam National University-Ho Chi Minh City, Ho Chi Minh City, Vietnam; 2 School of Business Administration, International University- Vietnam National University-Ho Chi Minh City, Ho Chi Minh City, Vietnam; 3 School of Economics, Finance and Accounting, International University- Vietnam National University-Ho Chi Minh City, Ho Chi Minh City, Vietnam; Zhejiang University of Technology, CHINA

## Abstract

This study examines the interplay between environmental regulations, organizational innovation, resilience, learning support, and performance in the Vietnamese business context. The paper explores the mutual interaction and influence among these variables. Additionally, it focuses on the indirect effects of organizational innovation and resilience, showing that organizational innovation mediates the relationship between environmental regulations and performance and resilience mediates the relationship between organizational learning support and performance. The study extends the framework of the dynamic capabilities perspective by demonstrating that dynamic capabilities enable organizations to adapt to and capitalize on stringent environmental policies. Our sample data come from 349 Vietnamese manufacturers and are analyzed using partial least squares structural equation modeling, which is effective for examining complex relationships and interactions among multiple variables. The results indicate that, although environmental regulations do not directly impact organizational performance, they do so indirectly through organizational innovation. The study also demonstrates the significant role of innovation and resilience in enhancing performance, yielding valuable insights for organizations that seek sustainable growth amid uncertainty. These findings lead to practical implications for developing strategies and policies that promote resilience, innovation, and learning, including a robust framework for addressing management challenges in emerging economies.

## 1. Introduction

The Doha Program of Action for developing countries in 2023 calls industrial activity the cornerstone of sustained economic development and one of the leading “drivers of prosperity” [1]. The 2023 industrial report from the United Nations Industrial Development Organization (UNIDO) emphasizes the importance of achieving industrial development while prioritizing environmental sustainability. The Environmental Performance Index in 2022 ranks Vietnam 178th among emerging and developing countries, suggesting that environmental issues may hinder the country’s economic development. In contrast to other governments in emerging markets, the Vietnamese government maintains significant economic control and, therefore, significantly shapes firms’ management practices [2], including environmental policies. Consequently, firms in Vietnam are incorporating environmental sustainability into their business operations and effectively managing their internal resources. Despite growing interest in the establishment of a positive relationship between environmental compliance and sustainable development, the existing literature indicates that enterprises have been unable allocate internal dynamic resources to comply with environmental regulations in order to enhance innovativeness and improve overall business performance.

Firms frequently encounter external disruption, such as natural disasters, political unrest, and pandemics. These disruptions can pose a significant threat to a firm, as they are typically unpredictable and out of its control [3]. Consequently, they have become increasingly concerned about their capacity for responding to various man-made crises and natural disasters [4]. In recognition of the growing significance of maintaining organizational resilience to these evolving challenges, firms seek ways to bolster their resilience.

Organizational resilience now encompasses not only the ability to recover from disruptions but also the innovative capacity to anticipate and create new opportunities [5, 6]. This wider perspective enables enterprises to effectively deploy their resources for overcoming current challenges and pursuing future opportunities [7]. Previous research has highlighted the importance of organizational resilience in enhancing business performance; however, few papers focus on the relationship between organizational learning support and resilience in specific business contexts, such as Vietnam. Moreover, the potential interaction effects of environmental regulation and innovativeness have not been thoroughly explored.

At the same time, a debate over the impact of environmental regulations on business competitiveness and productivity continues in the literature. Some studies demonstrate the positive role of environmental regulations [8–11], and others argue that these regulations might not enhance competitiveness or productivity[12–14]. This absence of consensus indicates the need for further investigation into the nuanced effects of environmental regulations. Additionally, although existing studies have found that innovation and resilience positively influence organizational performance, they often overlook their multidimensional nature [15, 16]. Consequently, further research is needed to determine the collective contribution of different forms of innovation to organizational success, thereby giving a more comprehensive explanation of their impact.

This study fills these research gaps by applying the theory of the dynamic capabilities view (DCV) to demonstrate that organizations adapt to external environments by being willing to learn about and build diverse networks [17, 18]. In short, organizations bolster their dynamic capacity for adjusting, responding to, enduring, and generating novel ideas so that they can flourish and succeed in this challenging setting and achieve sustainable development [10]. By extending the DCV, this paper shows that environmental regulations drive organizational innovation and improve performance [19]. It also examines the role of organizational resilience and learning support in enhancing organizational performance. This comprehensive approach enables a deeper understanding of how organizations can effectively navigate and thrive under dynamic and challenging business conditions.

Vietnam’s socialist-oriented market economy presents both opportunities and challenges for local businesses as they become integrated into the global economy [2]. Government support might facilitate trade flows and access to larger markets, but it also heightens competition between foreign companies and local enterprises. This competitive pressure drives local businesses to invest in innovation and sustainability in order to enhance performance and ensure their survival.

This study offers valuable insights into how businesses can achieve sustainable growth and success amid uncertainty and a dynamic business landscape. By filling the gaps in previous research, this paper deepens understanding on the interplay between environmental regulation, organizational innovation, resilience, and performance. To achieve these research objectives, we pose the following key questions:

RQ1. How do environmental regulations affect organizational innovation and performance?
RQ2. What impacts do organizational resilience and learning support have on organizational performance with interaction between environmental regulations and organizational innovation?

From a practical perspective, our findings help organizations develop strategic actions and policies that enhance resilience, innovation, and learning, leveraging the relationships among these factors to improve performance in areas such as economic outcomes, financial stability, customer satisfaction, and operations. Based on primary data from 349 Vietnamese enterprises, this study highlights the unique resource availability and management capacities of developing countries compared to those of Western and other developed countries. The proposed hypotheses are tested using the partial least squares–structural equation modeling (PLS-SEM) method. These results contribute to understanding management challenges in emerging economies, addressing the need for further research in this area.

The rest of this study is structured as follows. Section 2 reviews the theoretical background and proposes hypotheses. Section 3 presents the methodology. Section 4 reports the research results, and they are discussed in Section 5. Finally, theoretical contributions, practical implications, and conclusions with limitations are presented in Section 6.

## 2. Literature review

### 2.1. Theoretical background

The DCV concerns high-level routines employed by businesses in selecting management strategies from among several options, to generate particular valuable outputs, as well as their accompanying input flows [20]. In recent decades, the DCV has emerged as a significant perspective in business management [21]. It includes the identification and evaluation of organizational procedures and reorganizational resources through activities such as consolidation, acquisition, and release and the selection of a course of action to obtain strategic benefits.

The DCV is an extension of the resource-based view, which explains firms’ competitive advantage in a volatile and dynamic environment [22]. It encompasses three distinct actions. First, “sensing” is the capacity to identify, develop, and evaluate technological advancements in order to meet the demands of customers. Second, “seizing” is firms’ ability to acquire and effectively use the necessary resources to ensure customer satisfaction. Third, “reconfiguring” means activities that involve the recombination of traditional capabilities with diverse resources [23]. Likewise, Wilhelm et al. [24] see the competence of “learning” as having a role equivalent to that of seizing. Firms that want to ensure sustainable outcomes from their initiatives and practices will need to discover, exploit, and update their knowledge in order to survive in the market [25]. Based on this theoretical background, we extend the DCV to investigate the role of organizational innovation, environmental regulation, organizational learning, and organizational resilience in enhancing organizational performance.

### 2.2. Organizational innovation

Although innovation is a multifaceted concept, many definitions highlight the invention or development of something new or improved, which includes not just technological advances but also improvements in products, processes, and systems [26]. This comprehensive approach encompasses the creation of new solutions, the improvement of existing solutions, and the adoption and implementation of these innovations in organizational contexts. Innovation supports progress and competitiveness by stimulating creativity and the use of new ideas in a variety of areas, addressing emerging challenges and meeting evolving needs in various sectors [27]. Furthermore, the wide range of views in the literature has led scholars to explore various classification methodologies concerning innovation [28, 29]. Likewise, organizational innovation is directly correlated with organizational success, market share, and growth [30]. Moreover, several studies have emphasized the significance of innovation in sustaining corporate development and overall profit [31, 32] and in achieving a sustainable competitive advantage [33]. Therefore, organizational innovation is essential for attaining long-term success, gaining a competitive advantage, and enhancing productivity.

### 2.3. Environmental regulations

Environmental regulations are official policies aimed at protecting the environment. They play an indispensable role in doing so by limiting the damage caused by firms [34] and have a multidimensional impact on companies’ innovation behavior, embracing and interweaving technological, product, and system innovation, as well as the relationship among them [35]. These rules encourage businesses to develop new technology, alter goods, and revamp systems in order to satisfy compliance requirements. Beyond mere development, these innovations encompass the adoption and implementation of sustainable practices and technologies, ensuring that businesses not only innovate but also efficiently incorporate these advancements into their operations. This holistic influence creates a dynamic environment in which regulatory demands drive comprehensive and sustainable innovation, resulting in long-term environmental and financial benefits.

### 2.4. Organizational learning

Organizational learning is a dynamic process that generates new information for practical use and implementation, leading to the creation of fresh knowledge that can be used and shared in the future [36–38]. Moreover, it is routine based, which requires combining current knowledge with newly obtained external knowledge [21]. Organizational learning contributes considerably to organizational resilience by improving adaptive ability, knowledge management, creativity, employee empowerment, and collaboration networks [37]. Similarly, organizational learning is a management competency that is crucial for a firm’s success, as it enables them to enhance and improve their knowledge, skills, and technology [39].

### 2.5. Organizational resilience

Organizational resilience, in the context of an enterprise, refers to its ability to withstand significant disruptions in its operations caused by unforeseen, unexpected, or catastrophic events, enabling organizational systems to function beyond their intended limits without incurring significant losses [40]. The concept of organizational resilience has recently garnered heightened attention because the current environment is characterized by unpredictability and rapid change [3]. Moreover, organizational resilience encompasses the use of novel capacities not only to monitor potential risks and challenges but also to proactively generate new opportunities, going beyond simply restoration efforts [5, 6]. Overall, enhancement of organizational resilience enables firms to use their resources not only to address current obstacles but also to pursue potential opportunities [7]. In other words, organizational resilience is widely acknowledged as an important quality that increases the likelihood of organizational success. This capacity enables companies to successfully navigate and recover from interruptions, maintain continuous operations, and adapt to changing conditions. Firms that encourage resilience can sustain productivity, increase competitiveness, and ensure long-term success, significantly enhancing overall performance [41].

Building on previous studies, we adopt organizational resilience as a second-order construct that encompasses three first-order constructs: robustness, agility, and integrity [38, 42]. Robustness refers to a firm’s ability to withstand and absorb shocks without a significant decline in performance [43]. Agility emphasizes the capacity to adapt quickly to changing conditions and seize new opportunities, ensuring that a firm remains competitive and responsive [44]. Finally, integrity involves maintaining consistency and ethical standards across operations, which builds trust and sustains long-term relationships with employees [38, 43].

### 2.6. Organizational performance

Organizational performance refers to the effectiveness with which organizations achieve their goals, encompassing the quality of management practices and the extent to which they deliver value to customers and other stakeholders. It is a critical indicator of an organization’s ability to align resources, processes, and strategies to meet its objectives [45]. Organizational performance is not just about meeting targets; it also involves the capacity to adapt to changing environments, innovate, and sustain competitive advantage [46].

## 3. Hypothesis development

### 3.1. Organizational innovation and organizational performance

According to the DCV, organizations can integrate, build, and reconfigure internal and external competencies, which is essential for achieving and sustaining competitive advantage, especially in dynamic environments [23]. Organizational innovation, as a dynamic capability, enables firms to adapt to changing market conditions, meet evolving customer demand, and exploit new opportunities [47]. By embracing innovation, organizations can cater effectively to the needs of environmentally sensitive clientele while also enhancing efficiency and cutting costs. This, in turn, can improve overall performance [48, 49]. This theoretical perspective suggests that innovation is not merely an outcome but a crucial driver of superior organizational performance.

From the empirical research perspective, previous studies show a significant relationship between innovation capability and organizational performance [50, 51]. Similarly, some recent studies provide strong evidence of a significantly positive association between organizational innovation and business performance [52, 53]. By expanding the DCV framework, we demonstrate that organizational leaders who actively foster innovation typically achieve early-mover benefits and higher profitability because of their consistent generation of novel ideas, processes, goods, and services [54]. Building upon this theoretical and empirical groundwork, we propose our first initial hypothesis:

*Hypothesis 1*: *Organizational innovation has a significantly positive impact on organizational performance*.

### 3.2. Environmental regulations, organizational innovation, and performance

According to the DCV, to ensure that the results from their endeavors and practices are long lasting and that they can thrive in the marketplace, organizations must acquire, leverage, and refresh their knowledge. Therefore, firms comply with environmental regulations to obtain more opportunities for increasing their innovativeness [53, 55]. First, environmental regulations can stimulate entrepreneurial innovation. Moreover, government policies can strongly incentivize enterprises to embrace innovative practices that foster recycling, reuse, and reduction of waste and consumption [56]. Similarly, government subsidies are often seen as an effective tool in research and development (R&D) for alleviating financial limitations faced by corporations and address deficiencies in the market [57]. Hence, regardless of whether through motivation or coercion, legislation pertaining to the environment ultimately stimulates innovation by organizations in the long term [58].

Moreover, empirical studies indicate an absence of consensus about the impacts of environmental regulations on organizational performance. Some studies provide strong evidence of a positively significant relationship between environmental restrictions and performance [53, 59, 60]. The regulations not only enhance the financial advantages of green technology but also reduce manufacturing expenses for businesses [61]. But other studies find a negative relationship because environmental regulations do not increase production capacity and firm competitiveness [12–14]; instead, these regulations increase expenses and appear to be ineffective for expanding organizational operations and performance [62]. Based on these arguments, we propose the following hypotheses:

*Hypothesis 2*: *Environmental regulations have a positive impact on organizational innovation*.
*Hypothesis 3*: *Environmental regulations have a positive impact on organizational performance*.

### 3.3. Organizational learning, resilience, and performance

A firm that aims to achieve a desired level of performance needs to cultivate a culture of learning within it. This aligns with the DCV, which emphasizes the importance of a firm’s ability to integrate, build, and reconfigure internal and external competencies in order to deal with a rapidly changing environment and ensure long-term market success [25]. This can also be achieved by integrating current information and simultaneously obtaining new knowledge [63]. Moreover, firms that cultivate a culture of learning are better equipped to embrace innovation. As a result, they can capitalize on opportunities for technological advancement, which enhances organizational performance [64]. In addition, the integration of environmental learning dynamics into knowledge sharing can have a substantial influence on organizational performance [65]. Likewise, organizational learning is crucial for effectively implementing new methodologies, thereby enhancing organizational performance [66].

Additionally, organizational learning has a beneficial impact on organizational resilience through multiple channels [37]. This aligns with the principles of the DCV, which posits that dynamic capabilities, such as learning, are essential for building resilience and sustaining competitiveness [22]. First, firms that aim to improve their resilience so as to withstand challenges actively seek knowledge that enables them to promote adaptation, flexibility, and competency. These characteristics enable organizations to remain robust and competitive [37, 38]. Second, firms that prioritize learning are better positioned to capitalize on opportunities [37]. Similarly, learning facilitates the sharing of knowledge and the generation of novel ideas, which are crucial for developing resilience [67, 68], as well as helping firms develop their competencies [69]. Nevertheless, little empirical research has combined these two elements into a conceptual framework with organizational learning and resilience, which are pivotal features in the DCV and help a firm to achieve its desired outcomes. Therefore, we propose the following hypotheses:

*Hypothesis 4*: *Organizational learning support has a positive impact on organizational performance*.
*Hypothesis 5*: *Organizational learning support has a positive impact on organizational resilience*.

### 3.4. Organizational resilience and performance

The DCV suggests that an organization’s ability to adapt, integrate, and reconfigure internal and external resources in order to deal with rapidly changing environments is fundamental for achieving a sustained competitive advantage [22]. As an organizational capability, resilience plays a crucial role in this context. It enables firms to handle and adjust to unexpected situations, maintaining stability and performance both under normal conditions and during crises [7]. Firm effectiveness is influenced by resilience, both under normal conditions and during crises, as individuals at firms effectively handle and adjust to unexpected events [70].

In empirical research, resilience is regarded as a beneficial ability that has a positive impact on organizational performance [41]. Moreover, firms can achieve higher performance by enhancing their resilience, improving product quality, meeting market demand, and improving operational effectiveness. Likewise, resilience enables them to address obstacles, attain profitability promptly [19] and enhance business performance [7]. By connecting these arguments to the DCV, we show that resilience, which functions as an organizational capability, can improve performance. Our final hypothesis is as follows:

*Hypothesis 6*: *Organizational resilience has a positive effect on organizational performance*.

### 3.5. The mediating role of organizational innovation and resilience

Previous research has established a direct relationship between environmental regulations and organizational performance [53, 59, 60] as well as between organizational innovation and performance [52, 53]. However, few studies have explored organizational innovation as a mediator in this context.

Prior studies support a positive and direct relationship between organizational resilience and performance [7, 19] and between organizational learning support and performance [64–66]. Organizational resilience improves a company’s ability to endure interruptions and sustain high performance. Organizational learning promotes both ongoing improvement and innovation, which directly enhances performance. Nevertheless, the existing body of knowledge has a gap concerning the mediating function of organizational resilience in this dynamic context.

Finally, organizational innovation, driven by environmental regulations, can enhance performance significantly by fostering early-mover advantages and consistent innovation. Therefore, this paper posits that organizational innovation mediates the relationship between environmental regulations and performance. Similarly, by incorporating resilience as a mediator, this study extends understanding on how learning support influences performance, offering a more comprehensive model of organizational effectiveness. This approach clarifies the indirect impacts of learning on performance, emphasizing the critical role of resilience in converting learning into concrete business results. These propositions are articulated through the following hypotheses:

*H7*: *Organizational innovation mediates the positive relationship between environmental regulations and organizational performance*.
*H8*: *Organizational resilience mediates the positive relationship between organizational learning support and performance*.

**Fig 1:**
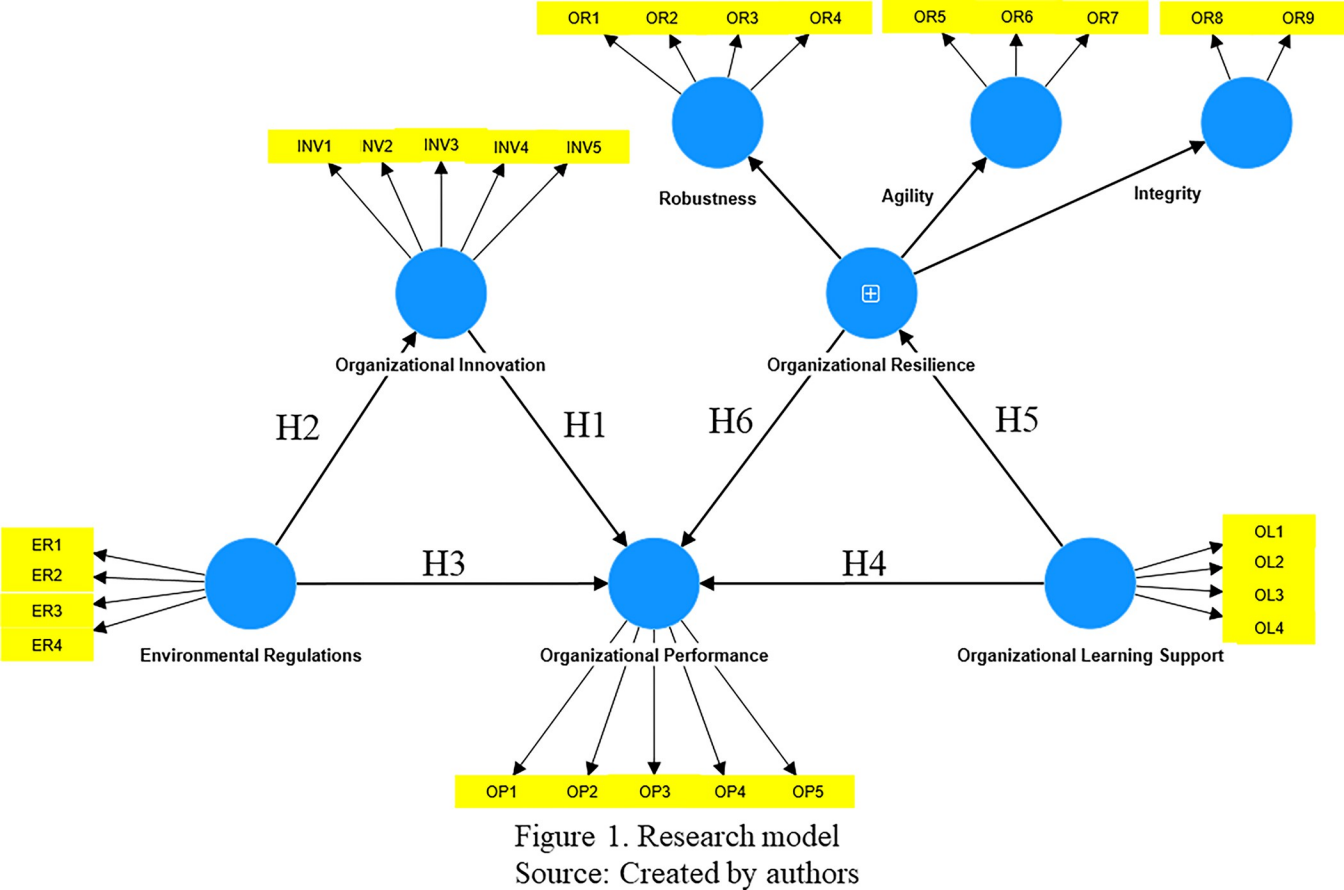
Insert around here.

Fig 1 depicts the theoretical research model and the hypotheses derived from the underlying theoretical framework of the DCV. In this model, organizational resilience is a second-order construct that establishes three first-order constructs: robustness, agility, and integrity. Our building of this second-order construct distinguishes this study from much of the literature reviewed earlier.

## 4. Methodology

### 4.1. Ethical statement

This study was conducted in compliance with the Ethical Principles of Psychologists and Code of Conduct outlined by the American Psychological Association. Every participant provided written informed consent in compliance with the Helsinki Declaration. The procedures used were approved by both the employee council of the participating organizations and the ethics committee of the Center for Public Administration (CFPA-RC-23-02-23).

### 4.2. Measurement

For this study, we created a well-organized questionnaire that included established scales and validated methodologies used in previous studies. To convey their level of agreement with each item on the scale, the respondents used a seven-point Likert scale, from 1 (strong disagreement) to 7 (strong agreement). This gave researchers valuable data about the respondents’ perspectives and thinking. The feedback from group discussions and the pilot test enabled us to make some adjustments in order to ensure the appropriateness of both the language and content.

All the measures of latent variables or constructs in the research model were adapted with minor or major modifications from previous studies. In particular, organizational innovation, with five items, was adapted from [30]. Environmental regulations, with four items, were adapted from [58]. Organizational learning support, with four items, was adapted from [37, 38]. Organizational performance, with five items, was adapted from [52, 53, 71]. Organizational resilience was established as a second-order construct with three first-order constructs: robustness (four items), agility (four items), and integrity (two items). All these items were adapted from [38]. Table 1 gives a thorough assessment of these constructs, organized based on their level of inheritance and offering vital information on their modification from previous studies.

**Table 1:** Measurements.

Constructs	Code	Modification
**Organizational Innovation**	**INV**	** **
Our company has a competitive mindset and can utilize new technology.	INV1	Major change
Our company attempts to become the first mover advantage in adding product value.	INV2	
Our company is willing to take risks when adopting new advanced technology.	INV3	
Our company understands and has expertise in using Information and communications technology (ICT) applications in the workplace.	INV4	
The current technological projects support innovation development.	INV5	
**Environmental Regulations**	**ER**	** **
The technology used in our company must strictly follow environmental guidelines and requirements.	ER1	Minor Change
Our company follows environmental standards for the enterprise pollutant emission intensity values.	ER2	
The government allocates a specific grant to businesses to develop technical advancements to enhance cleaner manufacturing.	ER3	
The government provides tradable licenses and incentives for pollution control to encourage enterprises to embrace innovation.	ER4	
**Organizational Learning Support**	**OL**	** **
Our company has attained and allocated new and relevant knowledge that provided a competitive advantage.	OL1	Minor Change
Members within the company have obtained certain critical skills and capabilities that could help obtain a competitive advantage.	OL2	
New knowledge shared within the company has a fundamental influence on the improvement.	OL3	
Our company is a learning organization.	OL4	
**Organizational Performance**	**OP**	** **
Our company can pursue sustainable development.	OP1	Minor Change
The company’s products or services have undergone a gradual improvement in their quality over time.	OP2	
The company had a strong reputation in its industry.	OP3	
The company’s customer appreciates the superior quality of its products or services.	OP4	
The company has witnessed a rise in its sales volume over the past three years.	OP5	
**Organizational Resilience (OR)-Second-Order Construct**	**OR**	** **
**Robustness First-Order Construct**	** **	** **
Our company maintains its upright stance and safeguard.	OR1	Minor Change
Our company succeeds in stimulating diverse solutions.	OR2	
Our company exhibits unwavering resilience to avoid defeat.	OR3	
Our company perseveres and remains steadfast in its course.	OR4	
**Agility First-Order Construct**	** **	** **
Our company acts quickly.	OR5	Minor Change
Our company develops alternatives to take advantage of negative situations.	OR6	
Our company promptly takes essential action when demanded.	OR7	
**Integrity First-Order Construct**	** **	** **
Our company fosters an inclusive workplace where all employees actively fulfill their responsibilities.	OR8	Minor Change
Our company effectively operates as a cohesive unit with all its personnel.	OR9	

Source: Created by the authors.

### 4.3. Sampling techniques

This study aims to determine the linkages between environmental regulations, organizational innovation, organizational resilience, organizational learning support, and organizational performance. To collect the data, we employed a sampling methodology based on surveys. In order to ensure that the measurements were well suited to the Vietnamese context, an English-language lecturer initially translated the questionnaire into Vietnamese and made some adjustments. Subsequently, the authors held three group discussions with seven directors from various manufacturers and three government executives serving on the administration boards of industrial zones. In response to input from participants, we modified the questionnaire to align it with the research setting in Vietnam. In addition, the authors conducted the pilot test with 40 participants and made minor adjustments to ensure the appropriateness of the interpretations within the research environment.

The sample selection received robust endorsement from the management boards of the industrial zones in Dong Nai, Binh Duong, and Tien Giang Provinces in Vietnam. They provided a comprehensive list of 980 prospective manufacturers, of which 550 were randomly selected to ensure a representative and unbiased sample. The data collection process from March to August 2023 included multiple follow-ups to maximize response rates and ensure data validity. Despite challenges, such as engaging busy executives and ensuring consistent responses, the process yielded 349 valid responses. These challenges were dealt with through persistent follow-up and clear communication, ensuring reliable and comprehensive data for analysis.

### 4.4. Analytical method

We evaluate the research model using PLS-SEM for several key reasons. First, PLS-SEM, a variance-based SEM method using a series of ordinary least squares regressions, is more adaptable than covariance-based SEM, making it suitable for use with complex models [72]. Second, our conceptual framework includes multiple dependent constructs, and PLS-SEM enhances predictive accuracy by optimizing these dependent variables locally [53]. Additionally, PLS-SEM can simultaneously represent numerous interactions and effectively address endogeneity issues, enabling the study to take a comprehensive analytical approach.

## 5. Results

### 5.1. Demographic characteristics

The data collection provided thorough information about the sample’s characteristics, which are listed in Table 2.

**Table 2:** Descriptive statistics.

Characteristics	Respondents (N = 349)	%
*Organizational size*		
Less than 200 employees	121	34.67
From 200–300 employees	135	38.68
Over 300 employees	93	26.65
*Education*		
Undergraduate Degree	245	70.20
Graduate Degree	104	29.80
*Position*		
Chairpersons	40	11.46
Board of Directors	68	19.48
Managers	241	69.05

Source: Created by the authors

### 5.2. Common method bias

Because we use a questionnaire to gather data from participants about both external and endogenous factors, the results might be influenced by common method bias (CMB). Before collecting the data, we took various measures to mitigate CMB, such as explicitly advising the participants that there were no objectively correct or incorrect responses and ensuring the anonymity of their answers [73]. To investigate the presence of collinearity in the data, we performed a statistical assessment of the complete collinearity. A value of the full collinearity variance inflation factor (FCVIF) below 3.3 indicates the absence of any problems regarding collinearity in the data [74]. The FCVIF for all our latent variables is below 3.3, as seen in Table 3, which shows that no CMB problems affected our data gathering.

**Table 3:** Reliability and validity.

Constructs	Code	Factor loading	α	CR	AVE	FCVIF
**Organizational Innovation**	INV1	0.810	**0.861**	**0.870**	**0.646**	**1.367**
	INV2	0.828				
	INV3	0.668				
	INV4	0.854				
** **	INV5	0.844	** **	** **	** **	** **
**Environmental Regulations**	ER1	0.743	**0.735**	**0.758**	**0.560**	**1.378**
	ER2	0.839				
	ER3	0.603				
** **	ER4	0.788	** **	** **	** **	** **
**Organizational Learning Support**	OL1	0.770	**0.735**	**0.756**	**0.553**	**1.856**
	OL2	0.801				
	OL3	0.649				
** **	OL4	0.744	** **	** **	** **	** **
**Organizational Performance**	OP1	0.800	**0.830**	**0.830**	**0.596**	**2.434**
	OP2	0.770				
	OP3	0.790				
	OP4	0.715				
** **	OP4	0.782	** **	** **	** **	** **
**Organizational Resilience-Second-Order Construct**			** **	** **	** **	** **
**Robustness-First-Order Construct**	OR1	0.833	**0.897**	**0.899**	**0.764**	**1.693**
	OR2	0.865				
	OR3	0.909				
** **	OR4	0.886				
Agility-First-Order Construct	OR5	0.899	**0.897**	**0.898**	**0.830**	**1.420**
	OR6	0.922				
	OR7	0.912	** **	** **	** **	** **
Integrity-First-Order Construct	OR8	0.930	**0.807**	**0.825**	**0.837**	**1.884**
	OR9	0.899				

Note: CR (composite reliability), AVE (average variance extracted), α (Cronbach alpha).

Source: Created by the authors.

### 5.3. Testing for validity and reliability

First, we need to evaluate the validity of a measured variable by observing the outer loading factors. An outer loading of 0.7 or higher is considered highly satisfactory [75]. Moreover, it is regarded as acceptable if an outer loading is above 0.5 [76]. According to Table 2, the load coefficient reached a minimum value of 0.603, indicating that the latent variables met the acceptable criterion.

Second, Table 3 presents the reliability and validity criteria. The Cronbach’s alpha and composite reliability (CR) values for all constructs are statistically significant and exceed the threshold of 0.7. The range of these values is from 0.735 to 0.897, providing evidence of the constructs’ reliability [77]. In addition, the average variance extracted (AVE) is employed to confirm the convergent validity of each construct. All the constructs have AVE values over the threshold of 0.5 [78], ranging from 0.553 to 0.837. Therefore, the constructs’ measurement scales are highly reliable and valid.

In addition, we use the Fornell-Larcker criterion [77] to assess the discriminant validity. Table 4 demonstrates that the square root of AVE for each construct (indicated in boldface) surpasses the interconstruct correlation that corresponds to it. This confirms that the model has adequate discriminant validity. Likewise, the heterotrait-monotrait ratio (HTMT) is used to confirm discriminant validity. Discriminant validity is considered satisfactory if all the HTMT values are below 0.9 [79]. Table 3 demonstrates that all the results are below the specified thresholds. Therefore, it is now even more evident that the constructs in this study have sufficient discriminant validity.

**Table 4:** Discriminant validity.

Heterotrait-Monotrait ratio (HTMT)							
Construct	(1)	(2)	(3)	(4)	(5)	(6)	(7)
Agility (1)							
Environmental Regulations (2)	0.650						
Integrity (3)	0.789	0.603					
Organizational Innovation (3)	0.805	0.651	0.741				
Organizational Learning Support (4)	0.734	0.604	0.722	0.696			
Organizational Performance (5)	0.792	0.564	0.803	0.780	0.877		
Robustness (6)	0.867	0.600	0.785	0.852	0.673	0.804	
Fornell-Larcker criterion							
Construct	(1)	(2)	(3)	(4)	(5)	(6)	(7)
Agility (1)	**0.911**						
Environmental Regulations (2)	0.533	**0.748**					
Integrity (3)	0.679	0.479	**0.915**				
Organizational Innovation (4)	0.709	0.525	0.621	**0.804**			
Organizational Learning Support (5)	0.617	0.457	0.569	0.575	**0.743**		
Organizational Performance (6)	0.684	0.449	0.658	0.663	0.709	**0.772**	
Robustness (7)	0.780	0.494	0.674	0.752	0.569	0.693	**0.874**

Notes: The square root of AVE is indicated in boldface.

Source: Created by the authors.

### 5.4. Structural equation model

**Fig 2:**
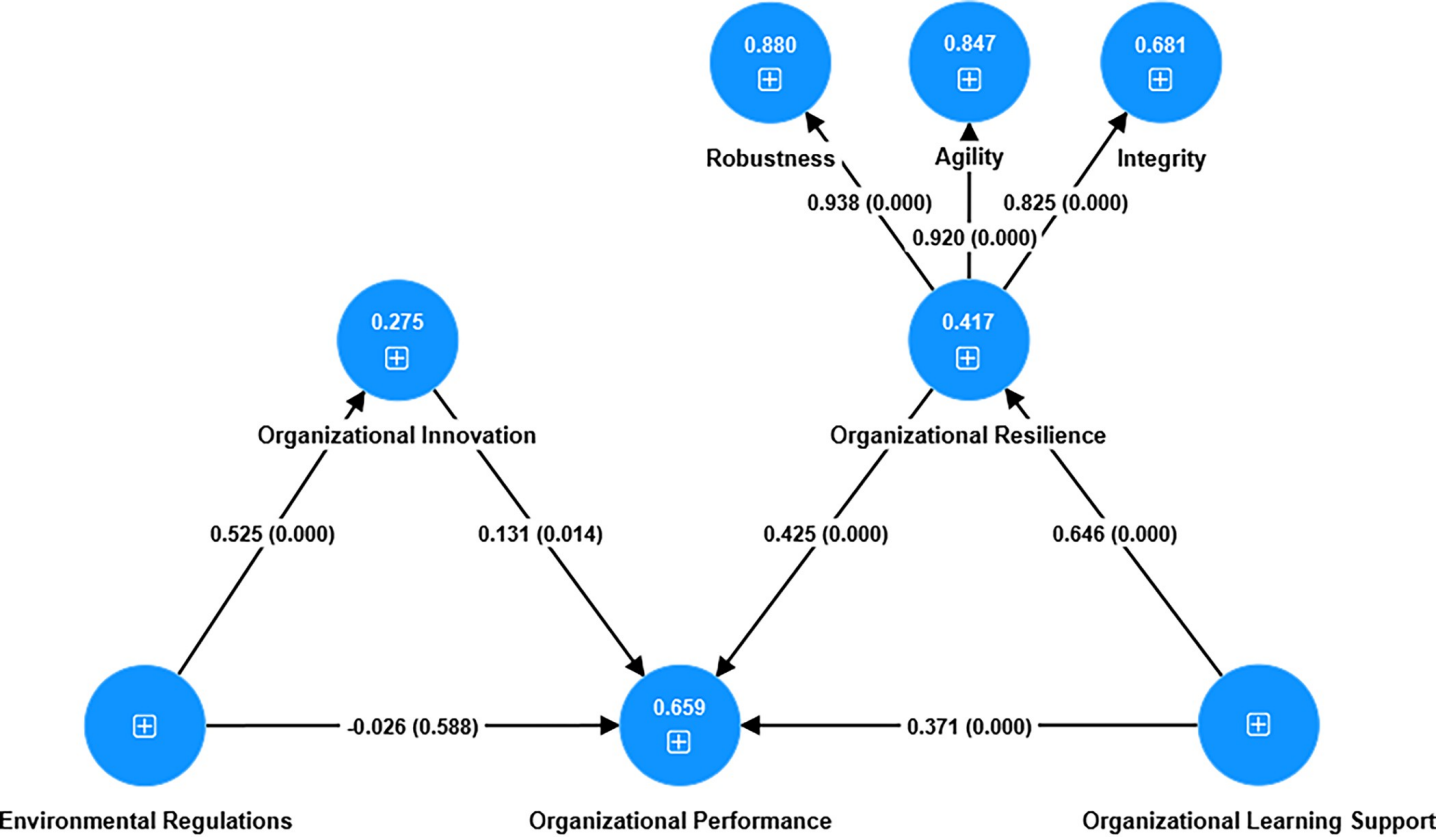
Insert around here.

Fig 2 illustrates the results from testing the hypotheses. The *R*^2^ values for organizational innovation, organizational resilience, and performance are 0.275, 0.417, and 0.659, respectively. These values are deemed to be weak, moderate, and substantial, respectively [80]. Table 5 also tests the hypotheses. All the hypotheses, except for H3, are accepted and have statistical significance (*p* < 0.05).

**Table 5:** Hypothesis testing.

**Direct effect**															
**Hypothesis**		**Coefficient**				**T- Values**			**P-value**			**Results**			
H1: INV *→* OP		0.131				2.463			0.014			Accepted			
H2: ER *→* INV		0.525				8.814			0.000			Accepted			
H3: ER *→* OP		-0.026				0.588			0.542			Not Accepted			
H4: OL *→* OP		0.371				7.268			0.000			Accepted			
H5: OL *→* OR		0.646				14.151			0.000			Accepted			
H6: OR *→* OP		0.425				6.156			0.000			Accepted			
**Mediation effect analysis**													
**Hypothesis**		**Type**		**Estimates**		**T-values**			**P-values**			**Remarks**	
H3: ER *→* OP		Direct		-0.026		0.588			0.542			Not Accepted	
ER *→* INV *→* OP		Indirect		0.069		2.328			0.020			Full mediation	
H4: OL *→* OP		Direct		0.645		12.087			0.000			Accepted	
OL *→* OR *→* OP		Indirect		0.272		5.739			0.000			Complementary	

Notes: INV: Organizational Innovation; ER: Environmental Regulations; OL: Organizational Learning Support; OP: Organizational Performance; OR: Organizational Resilience.

Source: Created by the authors.

In addition, we investigate the indirect effects of organizational innovation and resilience on the relationships between (1) environmental regulations and organizational performance and (2) organizational learning support and organizational performance. The mediation results are listed in Table 5, indicating that organizational innovation is a full mediator in the relationship between environmental regulations and organizational performance. Organizational resilience acts as a partial mediator in the relationship between organizational learning support and organizational performance.

## 6. Discussions

### 6.1. The role of environmental regulations in enhancing organizational innovation and performance

In today’s rapidly evolving business environment, organizations in emerging economies like Vietnam must simultaneously comply with stringent environmental regulations and drive innovation to maintain competitiveness. The Vietnamese manufacturing sector, pivotal to the nation’s economic growth, faces unique regulatory challenges that also present opportunities for innovation.

Manufacturing firms are under significant pressure to operate within a complex and evolving regulatory framework shaped by Vietnam’s transition to a market-oriented economy. They must adhere to global environmental standards and leverage these requirements to foster innovation. The relationship between regulatory compliance and innovation is particularly critical in Vietnam, where industrial growth is essential for economic development. Manufacturing firms must balance the immediate costs of compliance with the long-term advantages of sustainable practices, turning regulatory constraints into opportunities for competitive advantage.

This study, grounded in the Dynamic Capabilities View (DCV), investigates how environmental regulations, when combined with organizational resilience and learning support, influence innovation and performance in the Vietnamese manufacturing sector. The findings make a significant contribution to the literature on organizational innovation by illustrating that, in the context of Vietnam’s dynamic and evolving regulatory environment, firms can strategically leverage regulatory demands to drive innovation. This approach not only enhances organizational performance but also positions firms to better navigate and capitalize on regulatory challenges, turning them into opportunities for competitive advantage.

First, consistent with previous studies [50–53], this study provides strong evidence to support a significantly positive relationship between organizational innovation and performance. Innovation is a potent catalyst for overall performance as it empowers firms to adjust to evolving conditions, fulfill customer expectations, surpass rivals, and cultivate a culture of ongoing enhancement. Adopting innovation as a fundamental principle can greatly enhance the achievement and durability of organizational performance. Second, the findings show that environmental regulations have no direct effect on organizational performance. This result is inconsistent with previous studies [53, 59, 60]. It implies that the performance of a firm is not directly impacted by environmental regulations as its main objective is to ensure compliance, rather than improve performance. The upfront expenses related to adherence, such as the implementation of novel technologies and procedures, may overshadow the immediate advantages for performance. However, the effect of these regulations on performance is frequently influenced by organizational innovation and resilience. The DCV suggests that environmental rules can act as a catalyst for innovation, enhancing efficiency and creating new market prospects, ultimately leading to increases in performance.

However, environmental regulations have an indirect impact on organizational performance through organizational innovation. In other words, organizational innovation plays a full mediating role in connecting the relationship between environmental regulations and organizational performance. Therefore, firms are incentivized to implement sustainable practices and develop creative solutions because of environmental requirements. Environmental compliance can drive organizational innovation, which leads to better overall business performance. Finally, firms that prioritize sustainability in their operations not only maintain compliance with regulations but also position themselves for long-term success in a rapidly changing business landscape.

### 6.2. The role of organizational learning support in promoting organizational resilience and performance

Consistent with previous studies [63, 64, 66], this study confirms that support for organizational learning plays a vital role in improving both organizational resilience and performance. An environment that promotes ongoing learning cultivates a workforce that has expertise, flexibility, and the ability to adjust to changes effectively. Employees who work in an environment that supports learning are better prepared to deal with uncertainty and challenges, which greatly enhances the firm’s overall ability to bounce back from difficulty. Furthermore, organizational learning facilitates swift identification of and reaction to external change, such as shifts in market trends, technological improvement, and regulatory revisions. The ability to adapt plans and operations quickly is crucial for firms if they want to remain competitive and achieve optimal performance.

The results also emphasize that the impact of educational assistance on academic achievement is not only immediate but also influenced by resilience. The function of organizational resilience in mediating the relationship between learning support and performance shows that the direct impact of learning support on performance is enhanced when it also contributes to the development of firm resilience. This observation is consistent with and expands on other studies by showing that resilience is a crucial mechanism through which educational assistance leads to measurable enhancements in performance. Therefore, the study gives a detailed explanation of how learning support and resilience work together to enhance organizational performance, highlighting the significance of nurturing both factors for long-term success in a dynamic business environment.

## 7. Conclusions and implications

### 7.1. Theoretical contributions

The paper makes the following theoretical contributions as follows:

First, the initial DCV focuses primarily on traditional capabilities with diverse resources [23]. This study extends the DCV to include external factors, such as environmental regulations, and internal dynamic resources, such as organizational innovation, resilience, and learning support, to investigate their effects on organizational performance. Overall, our theoretical research strongly identifies the role of environmental regulations and learning support in enhancing organizational innovation and improving organizational performance.

Second, the study highlights that environmental regulations have no significant direct effect on organizational performance, but it has a significant direct influence on organizational innovation. Therefore, it accentuates how improving environmental regulations can stimulate R&D activities in order to devise innovative solutions. The theoretical models established in this study address the question of whether environmental regulations can either promote or hinder innovation efforts. In particular, our findings show that organizations may receive a specific grant or participate in incentive policies to improve technologies and achieve innovative solutions for better environmental protection.

Finally, this study establishes organizational resilience as a second-order construct by incorporating subdimensions for robustness, agility, and integrity, and the framework provides a more comprehensive understanding of organizational resilience. This integration enables a nuanced examination of how different aspects contribute to overall resilience. Moreover, gaining insight into the dynamic interaction among these subdimensions is essential for firms; those that can derive lessons from previous events and consistently enhance their strength, adaptability, and ethical standards will be more prepared to navigate future uncertainty.

### 7.2. Practical implications

First, this study highlights that Vietnamese manufacturing firms can comply with environmental regulations by strategically utilizing internal resources such as organizational innovation, resilience, and learning support. These resources not only enhance overall performance but also boost the firm’s creative capacity. Our findings offer practical guidance on implementing innovative strategies, including effectively engaging in R&D projects to gain a first-mover advantage in creating product value, embracing new technologies, and utilizing ICT applications to achieve sustainable development. Moreover, to maximize the benefits of these strategies, management teams must actively foster partnerships with government agencies. Such collaboration can help firms secure government support and incentives, particularly for upgrading technologies to meet environmental standards like ISO 14000, which is instrumental in controlling a company’s environmental footprint.

Second, our study provides a comprehensive analysis of organizational resilience in business contexts. The management team is generally expected to offer practical guidance on maintaining a firm’s reputation and sustainability, devising diverse solutions, maintaining resilience, acting quickly, encouraging employees to fulfill their responsibilities, and promoting innovative teams.

Third, practical strategies for supporting organizational learning involve cultivating a culture of ongoing enhancement through the implementation of learning platforms, frequent training programs that cover a wide range of critical skills and capabilities, and the promotion of knowledge-sharing activities. Implementing mentorship and coaching initiatives, fostering cross-functional collaboration, and integrating feedback loops into performance evaluations contribute to a comprehensive approach. In addition, offering external learning opportunities, employing learning metrics and analytics, and fostering a culture of continuous improvement guarantee that a firm remains adaptable and promotes continual employee growth.

Fourth, the management team should establish objectives based on practical considerations and dynamic resources that can readily be used by the company to enhance its ability to innovate and improve its performance in a swiftly evolving technical and regulatory environment. Our contributions facilitate the integration of theoretical concepts and practical applications, which improve the decision-making process and support sustainable development.

Finally, within the specific context of the Vietnamese manufacturing industry, the regulatory landscape presents both challenges and opportunities. Rapid industrial growth in Vietnam is a key driver of economic development but also brings significant environmental impacts. As leaders in the effort to meet global environmental standards, Vietnamese manufacturing firms must navigate a dynamic regulatory environment. This study underscores the importance of leveraging internal resources—not just to comply with regulations but to use them as a catalyst for innovation. By doing so, Vietnamese manufacturers can transform regulatory compliance into a strategic advantage, driving sustainable development and long-term competitiveness in an ever-evolving market.

### 7.3. Conclusions

This study extends the DCV framework by incorporating elements such as environmental regulations, organizational innovation, resilience, learning support, and performance in the Vietnamese business context. By doing so, it enhances understanding of how these factors interact in the current business environment. The findings significantly support most of our proposed hypotheses, with the exception of H3. Notably, organizational innovation fully mediates the relationship between environmental regulations and organizational performance, whereas organizational resilience partially mediates the relationship between organizational learning support and organizational performance. These insights are particularly valuable for Vietnamese businesses and policymakers, providing guidance on developing sophisticated and flexible external pressures to encourage environmental compliance and leveraging internal dynamic resources. This extended model enables management teams to navigate the complexity in their evolving resource environments with greater precision and purpose, fostering innovation and achieving long-term sustainability.

### 7.4. Limitations and future research goals

Although this study offers valuable insight, it has several limitations. First, the use of self-reported data may add bias or error because respondents might overestimate or underestimate their performance because of social desirability or recall errors. The inclusion of objective performance measures, such as financial records or third-party assessments, in future studies would strengthen the credibility of the findings. In addition, employing a combination of qualitative and quantitative methodologies to triangulate data sources could yield a more nuanced understanding of the phenomena investigated.

Next, our analysis fails to consider the potential moderating influence of other control variables, such as the age and size of the firm and the type of industry in which it operates, and demographic aspects, such as the educational background and degree of experience of its employees. These characteristics can have a substantial impact on the relationships examined in our model. Subsequent investigations should incorporate these variables to investigate their potential for mitigating the impacts of environmental legislation, organizational learning, and innovation on performance outcomes. Examining these moderating effects could yield a more profound understanding of how organizational environments can impact the execution and efficacy of resilience and innovation strategies.

Ultimately, although the study concentrates on particular variables and their interconnections, it fails to consider other aspects that may impact sustainable development. Further investigation should examine a wider selection of factors that could influence a firm’s effectiveness and long-term viability, including collective intelligence, organizational culture, security, and trust. Moreover, the study’s conclusions may not apply to other cultural or economic contexts than Vietnam. Future research should incorporate cross-cultural studies to gain insights into how diverse contexts impact the dynamics of environmental regulation, organizational innovation, and resilience. This will lead to the development of a framework that can be universally applied and the identification of strategies tailored to specific contexts, which could improve organizational performance and sustainability.

## Supporting information

S1 Data (XLSX)
